# Supplementary material for: Bithiophene-Based Donor–π–Acceptor Compounds Exhibiting Aggregation-Induced Emission as Agents to Detect Hidden Fingerprints and Electrochromic Materials
Source: Molecules. 2024 Dec 5;29(23):5747. doi: 10.3390/molecules29235747 (PMC11643829; doi:10.3390/molecules29235747)
Supplement: Supplementary file 1 [file molecules-29-05747-s001.zip › molecules-3318449-supplementary.pdf]

Supporting Information

Bithiophene-Based Donor–π–Acceptor Compounds Exhibiting Aggregation-Induced Emission as Agents to Detect Hidden Fingerprints and Electrochromic Materials

Patrycja Filipek, Magdalena Kałkus, Agata Szlapa-Kula and Michał Filapek*

Institute of Chemistry, Faculty of Science and Technology, University of Silesia, Szkolna 9, 40-007 Katowice, Poland; patrycja.filipek@us.edu.pl (P.F.); magdalena_kalkus1@op.pl (M.K.); agata.szlapa-kula@us.edu.pl (A.S.-K.)
* Correspondence: michal.filapek@us.edu.pl

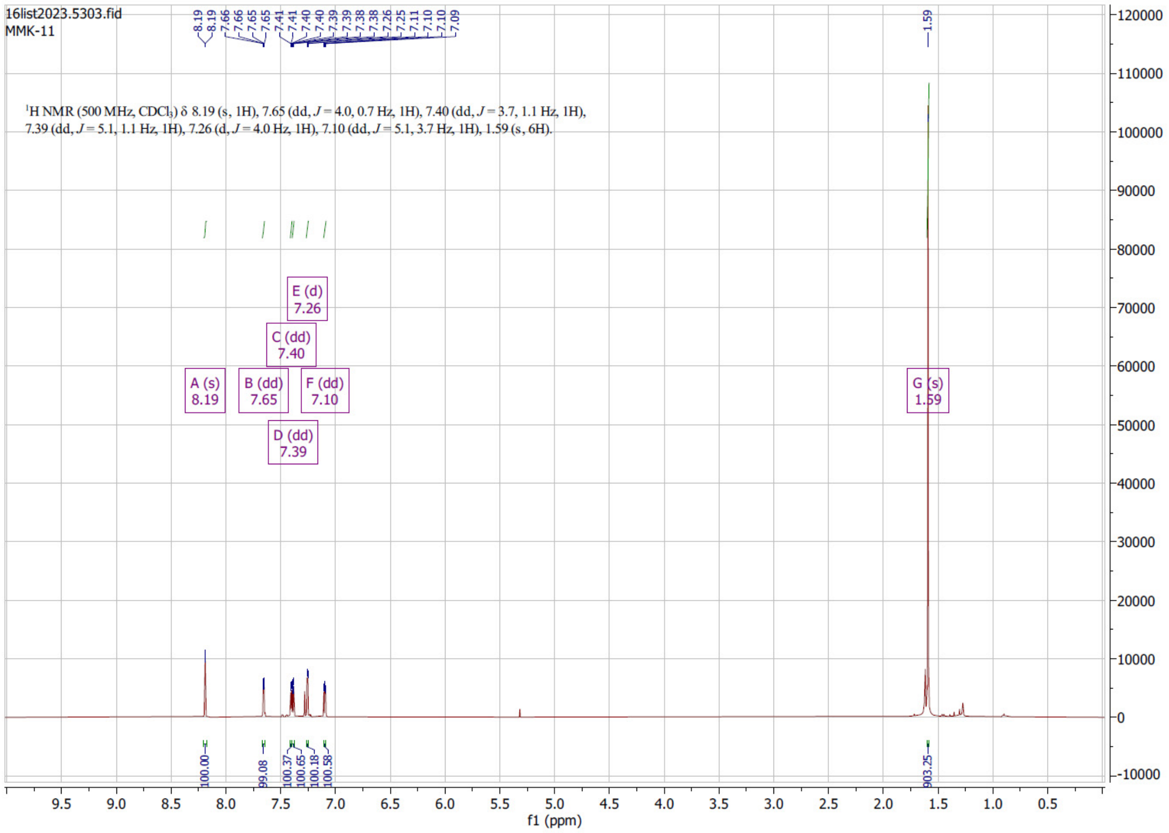

Spectrum S1.1H NMR spectrum of M3 (in CDCl3)

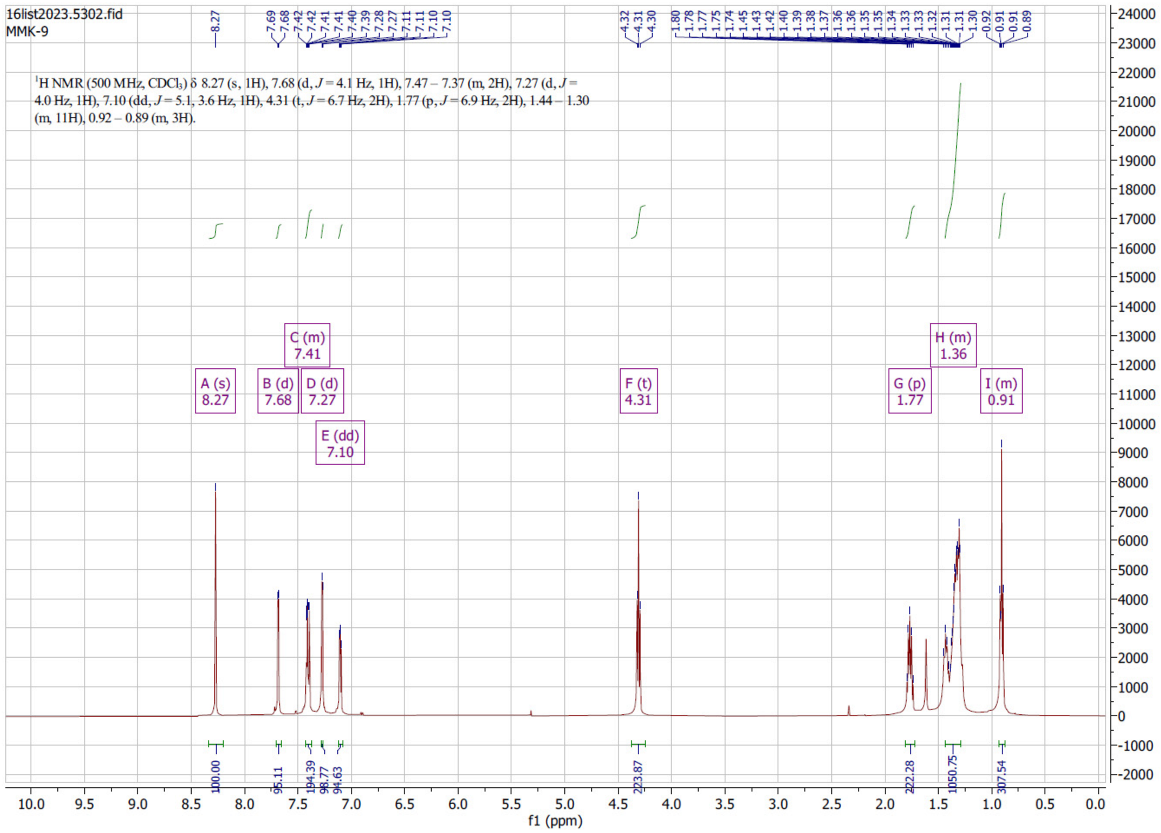

Spectrum S2. ^{1}H NMR spectrum of M4 (in $CDCl_3$)

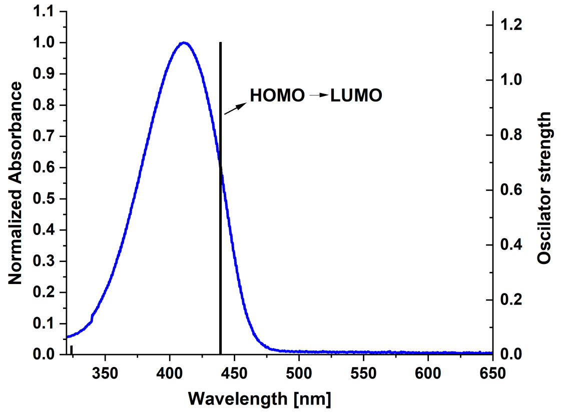

M1

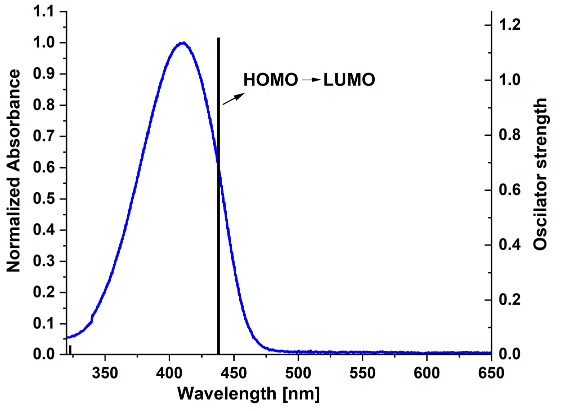

M2

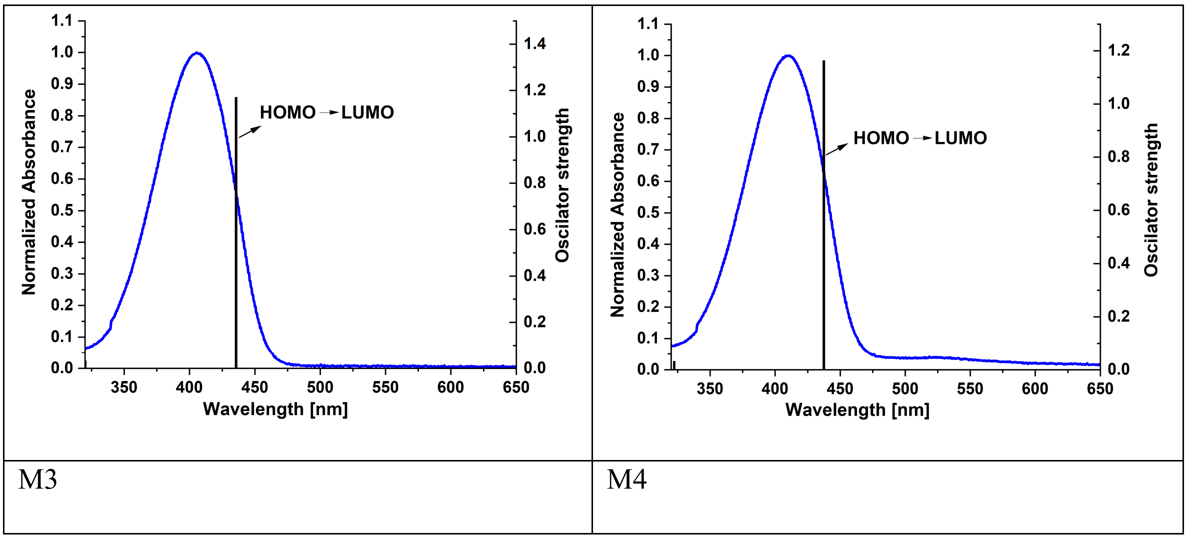

Figure. S1. Comparison of experimental (blue line) and theoretical (black line) absorption spectra.

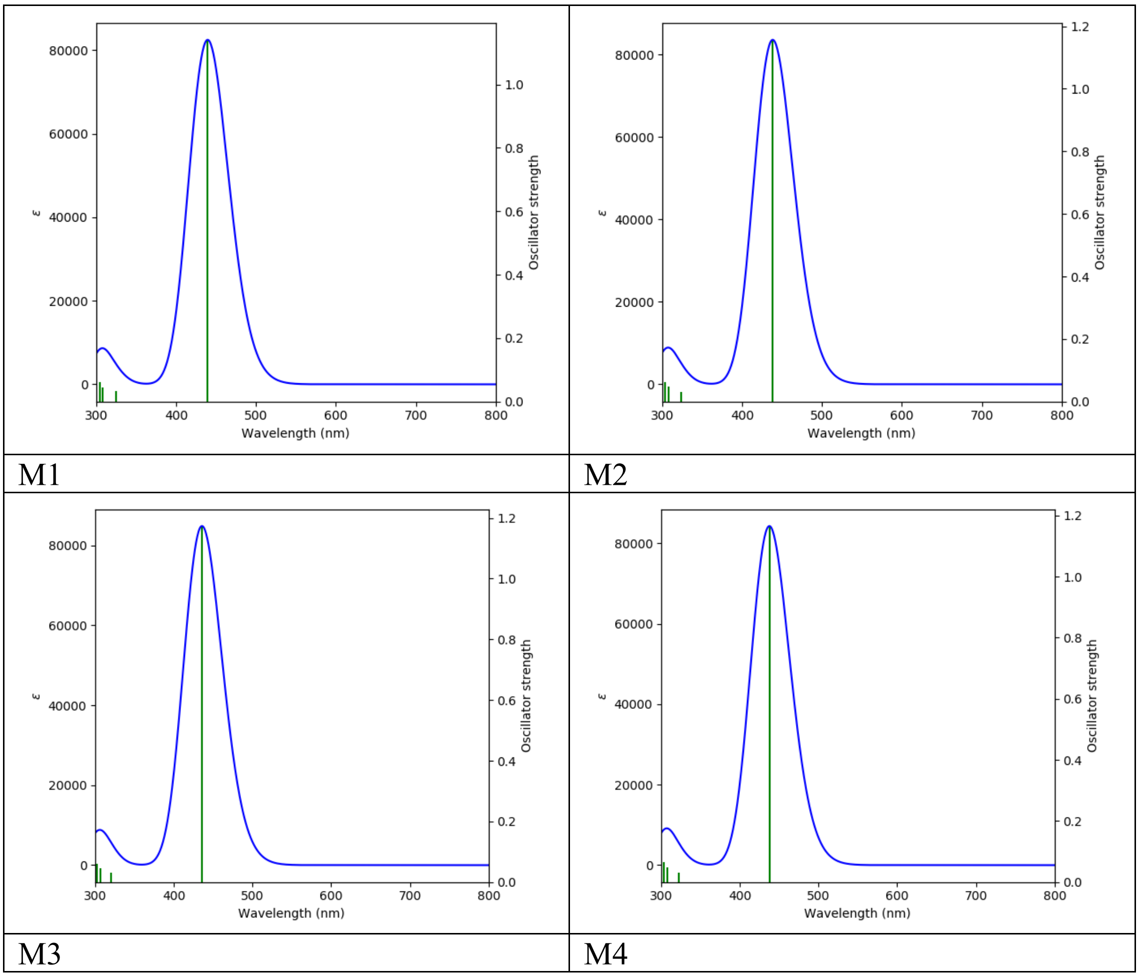

Figure. S2. Theoretical absorption spectra.

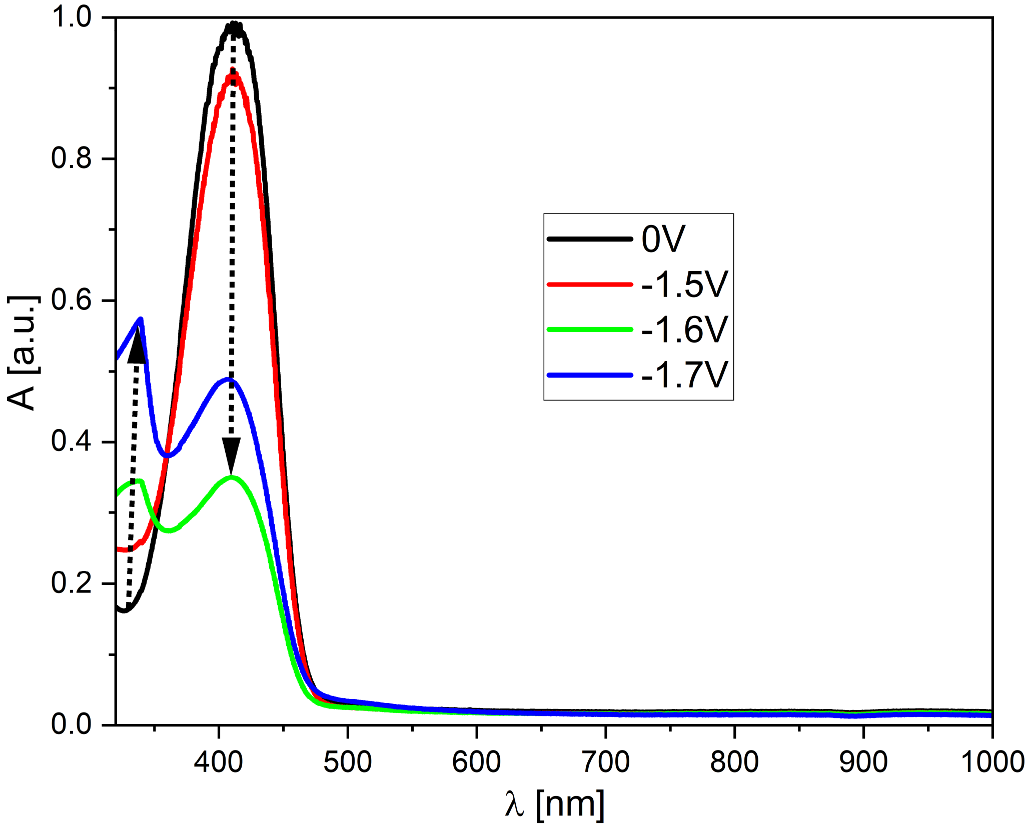

Figure S3. UV-Vis spectroelectrochemistry of the M1 in chloroform solution (c=1x10^{-5} mol/L, as an inset on each graph, all potentials vs Fc/Fc^{+} redox couple).

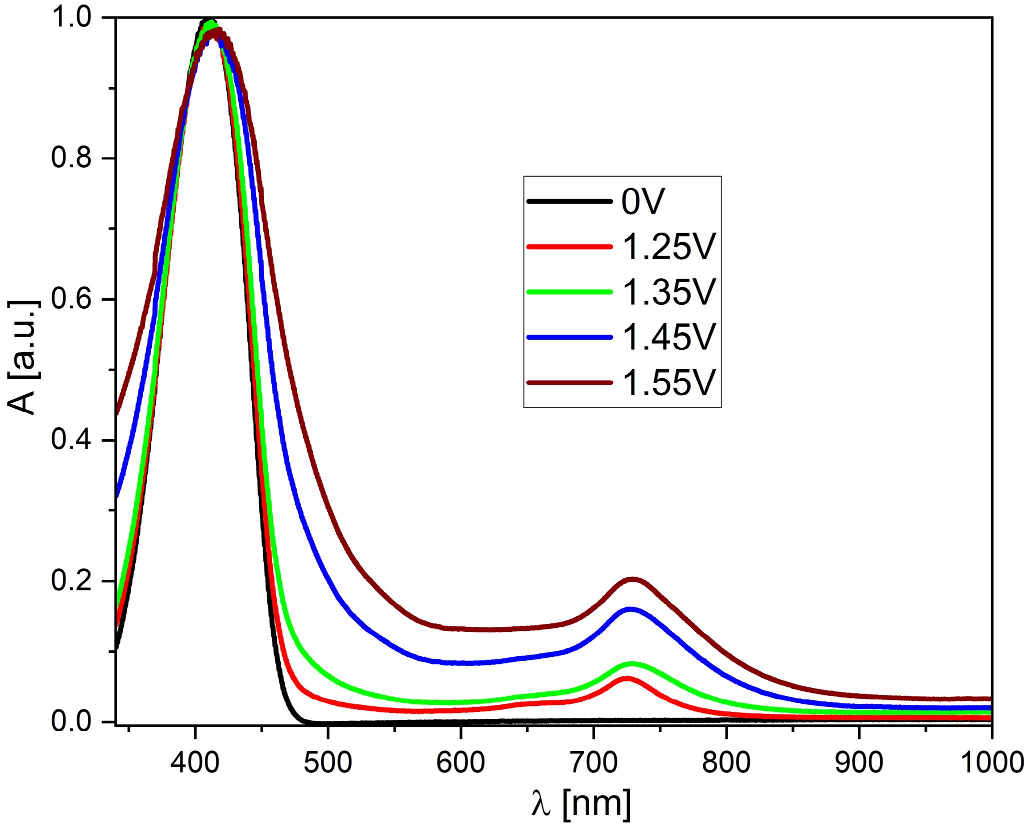

Figure S4. UV-Vis spectroelectrochemistry of the M4 in chloroform solution (c=1x10^{-5} mol/L, as an inset on each graph, all potentials vs Fc/Fc^{+} redox couple).

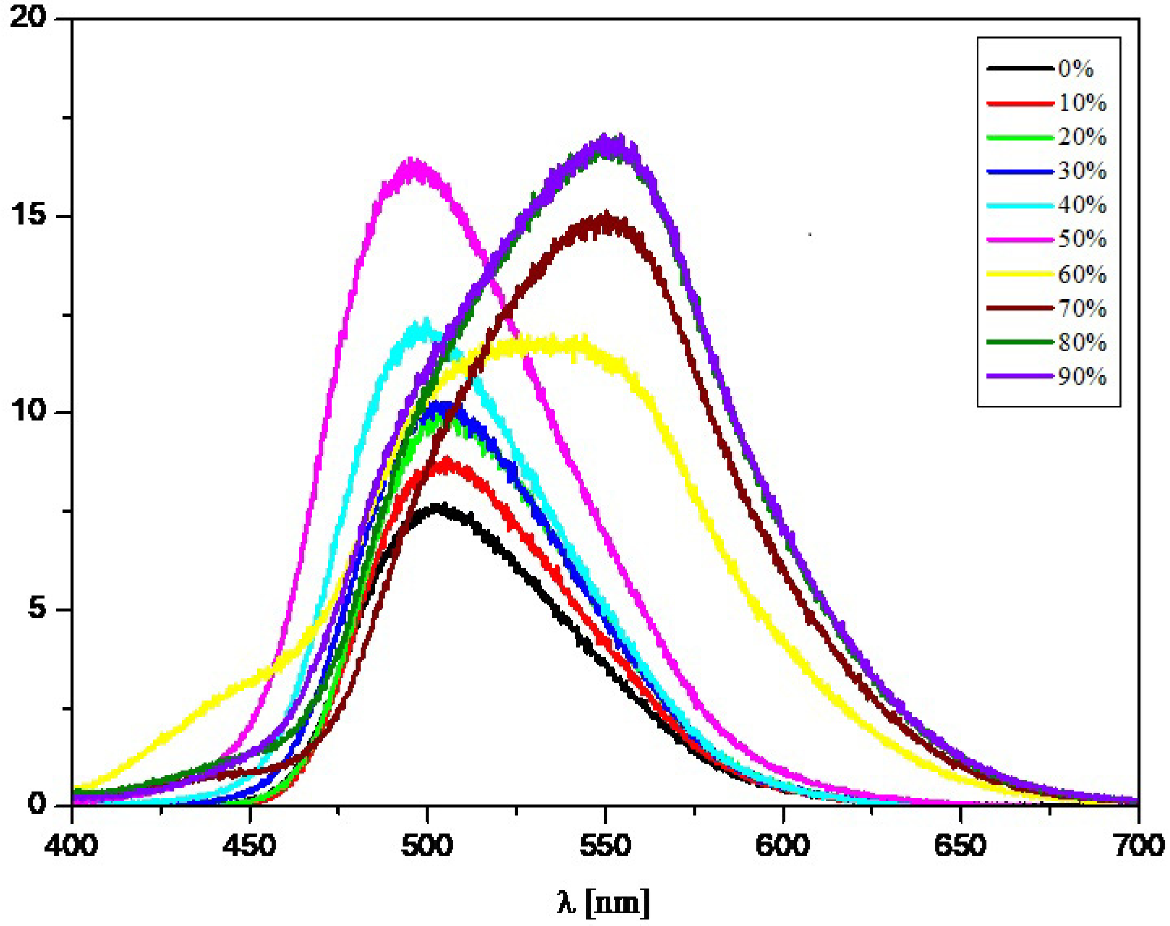

Figure S5. Emission spectra of **M3** in the mixtures of DMF and water (c=1x10^{-5} mol/L)

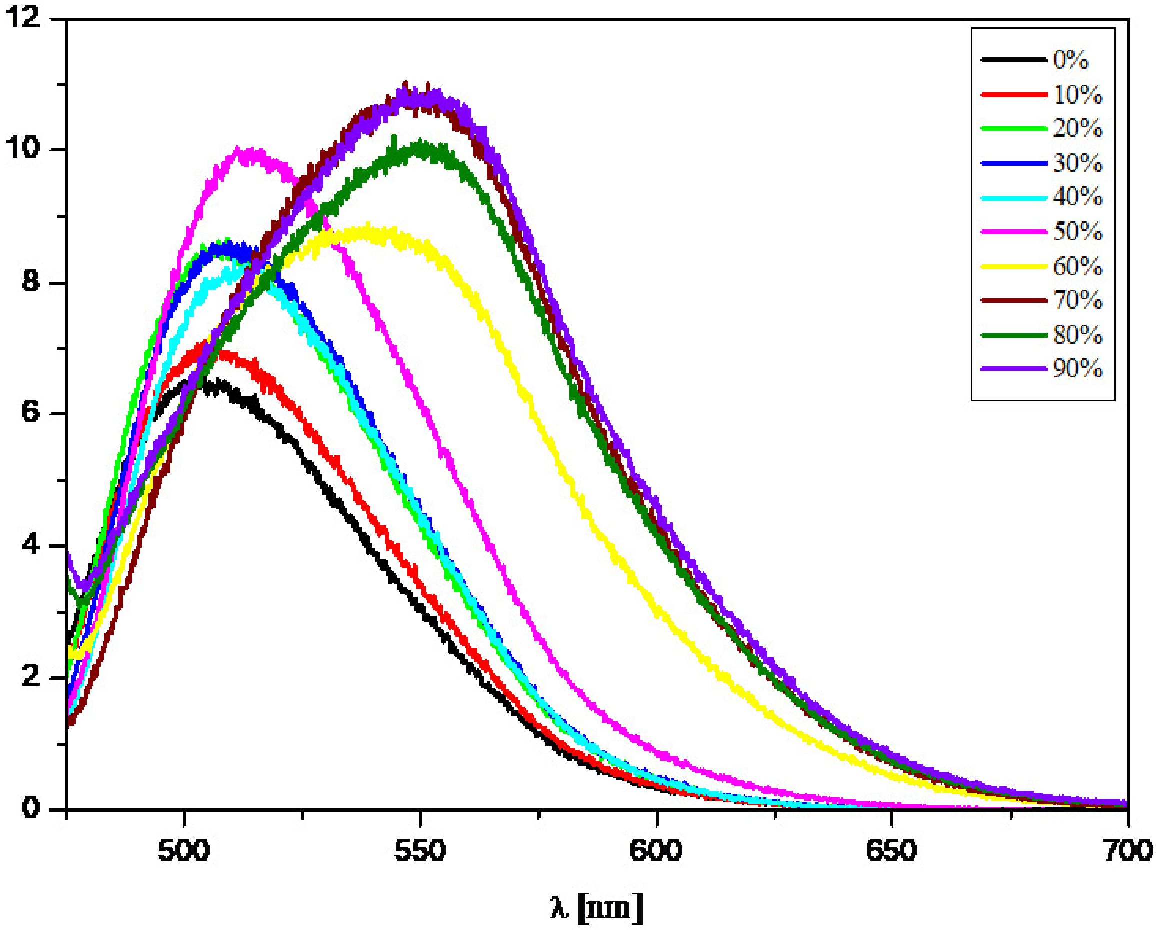

Figure S6. Emission spectra of **M4** in the mixtures of DMF and water ($c=1x10^{-5}$ mol/L)
